# A low power and low ripple CMOS high voltage generator for RFID transponder EEPROM

**DOI:** 10.1371/journal.pone.0225408

**Published:** 2020-02-05

**Authors:** Labonnah Farzana Rahman, Mohammad Marufuzzaman, Lubna Alam, Lariyah Mohd Sidek, Mamun Bin Ibne Reaz

**Affiliations:** 1 Institute for Environment and Development, Universiti Kebangsaan Malaysia, Bangi, Malaysia; 2 Institute of Energy Infrastructure, Universiti Tenaga Nasional, Kajang, Selangor, Malaysia; 3 Department of Electrical, Electronic and Systems Engineering, Faculty of Engineering and Built Environment, Universiti Kebangsaan Malaysia, Bangi, Malaysia; University of Science and Technology Beijing, CHINA

## Abstract

A high-voltage generator (HVG) is an essential part of a radio frequency identification electrically erasable programmable read-only memory (RFID–EEPROM). An HVG circuit is used to generate a regulated output voltage that is higher than the power supply voltage. However, the performance of the HVG is affected owing to the high-power dissipation, high-ripple voltage and low-pumping efficiency. Therefore, a regulator circuit consists of a voltage divider, comparator and a voltage reference, which are respectively required to reduce the ripple voltage, increase pumping efficiency and decrease the power dissipation of the HVG. Conversely, a clock driving circuit consists of the current-starved ring oscillator (CSRO), and the non- overlapping clock generator is required to drive the clock signals of the HVG circuit. In this study, the Mentor Graphics EldoSpice software package is used to design and simulate the HVG circuitry. The results showed that the designed CSRO dissipated only 4.9 μW at 10.2 MHz and that the phase noise was only -119.38 dBc/Hz at 1 MHz. Moreover, the proposed charge pump circuit was able to generate a maximum VPP of 13.53 V and it dissipated a power of only 31.01 μW for an input voltage VDD of 1.8 V. After integrating all the HVG modules, the results showed that the regulated HVG circuit was also able to generate a higher VPP of 14.59 V, while the total power dissipated was only 0.12 mW with a chip area of 0.044 mm^2^. Moreover, the HVG circuit produced a pumping efficiency of 90% and reduced the ripple voltage to <4 mV. Therefore, the integration of all the proposed modules in HVG ensured low-ripple programming voltages, higher pumping efficiency, and EEPROMs with lower power dissipation, and can be extensively used in low-power applications, such as in non-volatile memory, radiofrequency identification transponders, on-chip direct current DC-DC converters.

## Introduction

A radiofrequency identification (RFID) transponder is a chip or a small circuit board coupled to an antenna [1]. A typical RFID chip mainly contains three blocks: an analog block, logic and memory. To store data in the readerless RFID transponder, a small amount of nonvolatile memory (NVM) should also be embedded [2]. Depending on the device functionality, the tag memory may consist of read-only memory (ROM), random access memory (RAM), NVM such as electrically erasable programmable read-only memory (EEPROM) or flash memory and data buffers [3–4].

Many researchers attempted to develop EEPROM using a standard complementary metal-oxide semiconductor (CMOS) logic process because it is associated with low cost and low power advantages [5–8]. However, the N-type metal oxide semiconductor (NMOS) tunneling junction or the single-ended memory cell architecture with a too-thin oxide results in inadequate maintenance and endurance features [5–6]. Moreover, the single-ended memory cell architecture has a large area/bit and consumes considerable power as each bit cell contains its own high-voltage switch [7–8]. To generate high voltages, an internally regulated high-voltage generator (HVG) circuit, such as a high-voltage generator regulated by voltage doublers, or a charge pump (CP) circuit is required [9]. With the features of high-energy efficiency, small area, low-power consumption and regulated voltage generation, the HVG is a key module in an EEPROM. It is usually applied to the EEPROM in RFID transponders, direct current DC-DC converters and power management chips, to write or to erase floating-gate devices [10–13].

The prerequisites for low power and low-noise internal HVG have been gradually increasing owing to the escalating demand for robust and efficient RFID transponder EEPROM. To meet these demands, conventional power management units or HVG have to be redesigned with a stable output voltage for long-distance accessibility. The main challenges for system-on-chip (SOC) designers include the reduction of the power dissipation and ripple-free stable output of an integrated system in the CMOS process. A significant effort has been expended to the development of on-chip low-power and low-ripple HVG techniques [14–15].

In the RFID transponder, the EEPROM write/erase operations are driven by the internal HVG, which comprises of CP, current-starved ring oscillator (CSRO), non-overlapping clock generator (NOC_Gen), bandgap voltage reference (BGR), comparator, and voltage divider circuits. Moreover, each component has some constraints, which need to be considered while designing the HVG. For example, the CSRO should have low-phase noise and operate at high frequencies while the propagation delay and the settling time needs to be lower in NOC_Gen. The BGR should have a steady reference voltage and a small power-supply rejection ratio, while the comparator should have a high gain and a low-offset voltage with lower noise. Additionally, the CP circuit must have a small output noise, low-power dissipation, and a high-pumping efficiency with fast start-up time. In HVG, the module with the largest power dissipation is the CP circuit, which requires unique design challenges in terms of power efficiency, device reliability, driving capability and boosted performance. CP circuits with lower power efficiency limit the benefits of the boosted voltage on a chip, so it is desirable to increase the pumping efficiency not only in battery-powered devices but also in other applications with common supply voltages. Conversely, it is a challenging task for the designers to design a regulated CP circuit with a steady-state output voltage, low-power dissipation and minimum silicon area. The start-up time affects the functionality and the performances of all the supporting components of HVG too as faster start-up times reduce the CP energy dissipation upon and improve the overall efficiency. In addition, the output voltage ripple is a critical design specification, which degrades the performance of the overall high-voltage generation process. In particular, the ripple at the output of the CP circuit has a negative impact on sensitive analog circuits, such as the BGR, comparator and the control components. Finally, integrating all the HVG modules on a single die is another critical task for the researchers to reduce footprint and cost, which eventually helps the successful write/erase functionality of the RFID transponder EEPROM.

In this research, an HVG circuit is proposed which employs the clock driving circuit CSRO, the CP circuit along with a voltage regulator to reduce the power consumption, to increase pumping efficiency and decrease the ripple voltage from the output voltage. The Silterra 0.13 μm CMOS process is utilized to design and verify the proposed HVG circuit. This comparative study proves that the proposed HVG circuit scheme successfully achieves lower power dissipation, increases pumping efficiency and reduce voltage ripples compared with other recently published research studies.

## Materials and methods

**Fig 1:**
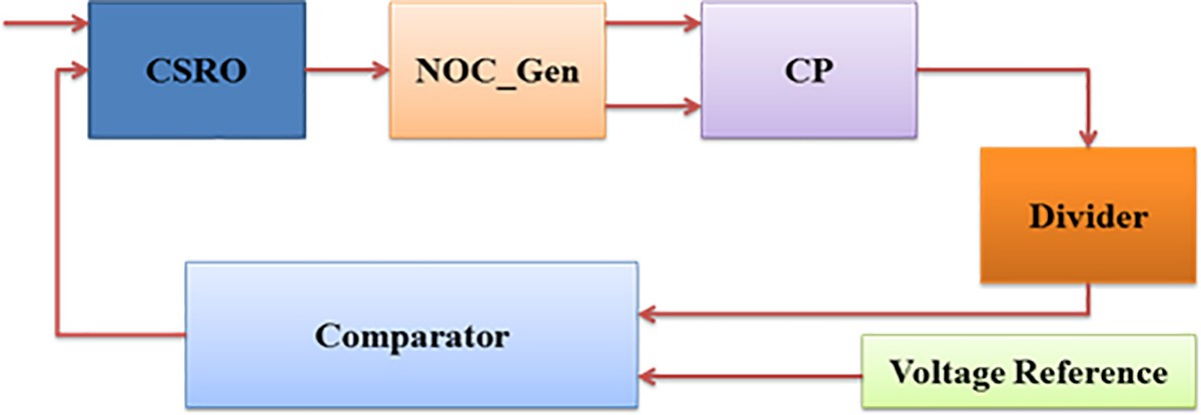
Block diagram of the proposed HVG.

An HVG is one of the most important components in RFID transponder EEPROM as it provides a boosted output voltage from the low supply voltage for write/erase operations. HVG performance greatly affects the overall performance of the EEPROM. To perform a write or erase operations in EEPROM, a CP circuit output voltage (VPP) need to be more than 14 V, which is higher than the reference voltage produced from a voltage reference circuit like the BGR [14]. Main parameters of an HVG are clock frequency, output voltage gain, ripple voltage, start-up time, power dissipation, pumping efficiency, chip size etc. Therefore, among all the currently available IC processes, the implementation of an HVG in CMOS is very much desirable for low power applications. Moreover, the popularity of CMOS is higher due to the ease in designing circuits with minimal power dissipation. In the interest of minimizing size and cost, a charge-pump, which requires only capacitors and integrated switches, is used as the primary voltage conversion mechanism. In order to function properly, the CP circuit needs to integrate with a voltage regulator to generate a feedback control voltage. The design of a high-efficiency HVG mostly depends on its feedback control circuit. Thus, the complete HVG is composed of a clock generation circuitry (CSRO and NOC_Gen), the CP circuit, and feedback control/regulator (capacitive divider, comparator, and BGR) circuit as shown in Fig 1

### Current starved ring oscillator

**Fig 2:**
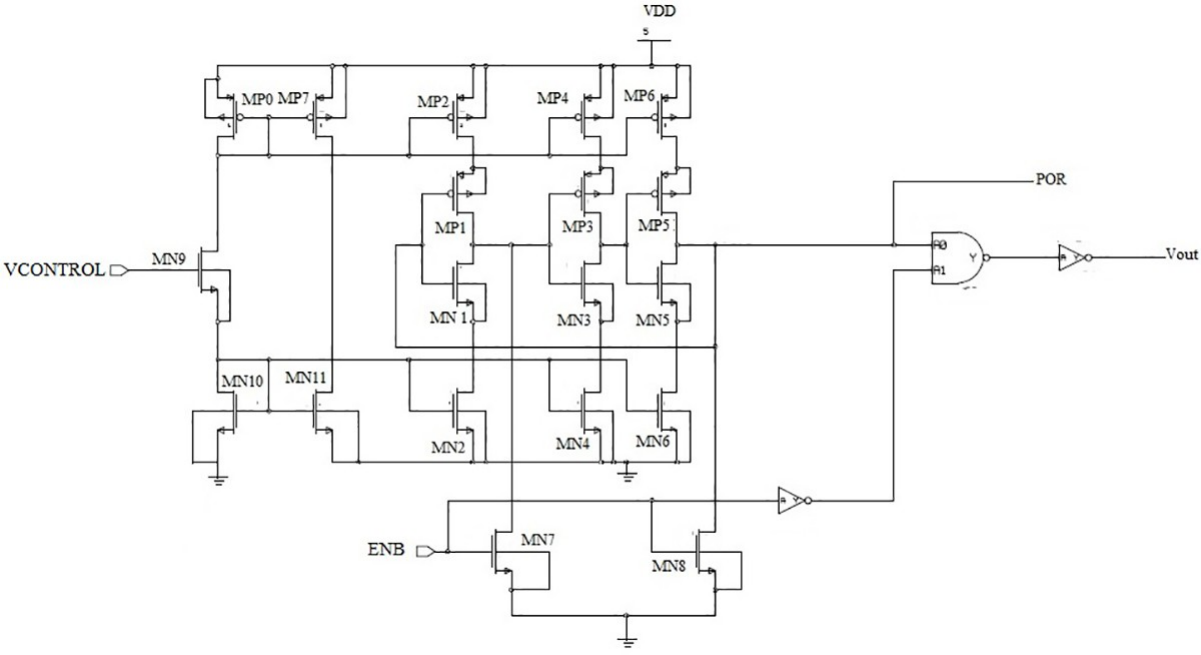
Schematic diagram of the current-starved ring oscillator (CSRO).

In this research, a three-stage CSRO is utilized, as shown in Fig 2. As shown, the transistors MN1/MP2 are used to act as an inverter in this design [16]. Conversely, MN2/MP2 acts as the mirror current source to limit the current flow through the inverter MN1/MP1. MP0, MP7, MN9, MN10, and MN11, are required to construct the biasing circuitry for the oscillator. In this design, MP7/MN11 has an equal drain current, which can be controlled by the input voltage VCONTROL, and is mirrored to each level of the oscillator. In this design, a power-on-reset (POR) circuit is embedded within the main circuitry to generate the reset signal for the chip. Moreover, when a power failure occurs from a certain level, this POR signal disconnects the chip. In this research, to measure the magnitude of VDD against a required threshold, the POR circuit consists of a NAND gate and a delay element. When the required threshold value is lower than the power supply voltage, a command signal is generated by this POR circuit to enable the overall chip functionality. Moreover, a delay element becomes active when the supply voltage is not in the set mode, and the reset button is set to the disable mode. Therefore, the overall frequency of the proposed oscillator can be defined by the following equation.

$$f=\frac{1}{{NT}_{D}}=\frac{I_{D}}{{NC}_{equ}V_{DD}}$$ (1)

where, *N* is the stage number, *T*_*D*_ is the delay, *C*_*equ*_ is the single-stage output equivalent capacitance, and *V*_*DD*_ is the supply voltage.

### Charge pump circuit

**Fig 3:**
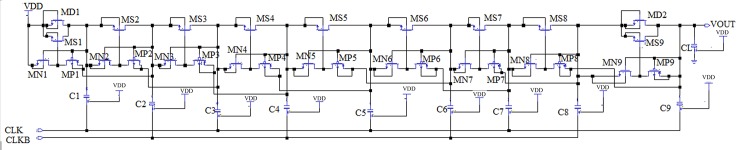
Schematic diagram of the proposed CTS based CP circuit.

At present, the CP circuits are suffering from output voltage loss, ripples, and lower pumping efficiency, owing to diode-connected topologies [17]. Therefore, several types of research studies have been conducted with the use of a new scheme of charge transfer switch (CTS’s), which removed the diode configuration. However, owing to the parasitic capacitance effect in each stage of the CTS, still voltage loss occurs at lower pumping efficiency levels. Therefore, in this research, a novel dynamic CTS scheme is utilized to develop the CP circuit. The schematic of this novel topology is shown in Fig 3.

Fig 3 shows that one diode-configured metal oxide semiconductor field-effect transistor (MOSFET) MD1 is required to initialize the voltage from the supply voltage VDD. Conversely, another diode-configured MOSFET MD2 is connected to the output stage. However, not all these MOSFETs are required to control the CTSs. To control the switches, CTSs (MS1–MS9) are utilized in this design to transmit the charges from the first to the last stage using a backward controlling method. Moreover, in this design, NMOS transistors are utilized in all stages to reduce the substrate current given that the use of P-type metal oxide semiconductor (PMOS) transistors in CTS schemes create large substrate currents. Not all CTS switches (i.e., from MS1–MS9) are completely turned “OFF” during the CTS. Therefore, extra controlling transistors (MN’s and MP’s) are employed in this design. Transistors MN1–MN9 are required to turn OFF all CTSs (MS1–MS9) during the charge transfer process. Furthermore, the pumping efficiency is increased at every stage, as all CTSs are turned OFF completely to prevent the reverse charge sharing phenomena. Moreover, all MP1–MP9 switches are required to turn “ON” all the CTSs to prevent latch-up. To boost up the charges, two clock signals, CLK and CLKB, are required, which are out of phase but have the same amplitude (V_clk_) as VDD, as shown in Fig 1. Finally, a diode-connected output stage has been employed in this design to increase the voltage gain or efficiency. The sizing of all transistors determines the total performance of the circuit. The operation method of this proposed CTS CP circuit is described with the second stage as an example. During the pumping stages, when the clock signal CLK = 1 and the anti-phase clock signal CLKB = 0, the gate of MS2 is turned ON completely, and it holds the value of VDD as the pass transistor MN2 is OFF and MP2 is ON. At this time, the threshold voltage V_th_ of MS2 becomes zero, and holds the value of VDD to node 2. Conversely, when CLK = 0 and CLKB = 1, the pass transistor MN2 is turned ON and MP2 is turned OFF. Therefore, the gate voltage of MS2 becomes zero, which turns OFF MS2 completely. In this manner, the entire CP circuit feeds back the charges from one stage to the next. In this topology, to overcome the threshold voltage drop at each stage, a zero V_th_ MOSFET is implemented, which is one of the advantages of this scheme.

Usually, V_clk_ is set to acquire the same voltage level as the normal V_DD_. The voltage fluctuation of each pumping node can be expressed as,

$$\Delta V\approx V_{clk}=V_{DD}$$ (2)

Hence, the output voltage of the N-stage CP circuit can be expressed as

$$V_{out}=N.(V_{DD}-V_{D})$$ (3)

The number of stages determines the power efficiency because of V_out_ and VDD, which are fixed by the specific CMOS process [16]. Compared with the initial Dickson CP circuit in which the diodes have zero V_t_, the power efficiency in the proposed CTS-based CP circuit is improved by VDD/ (VDD–V_t_) according to the following equation:

$$\eta_{power}=\frac{V_{out}.I_{out}}{(VDD-V_{t}).I_{in}}=\frac{V_{out}}{\left(N+1)(VDD-V_{t}\right)}$$ (4)

### Voltage regulator

**Fig 4:**
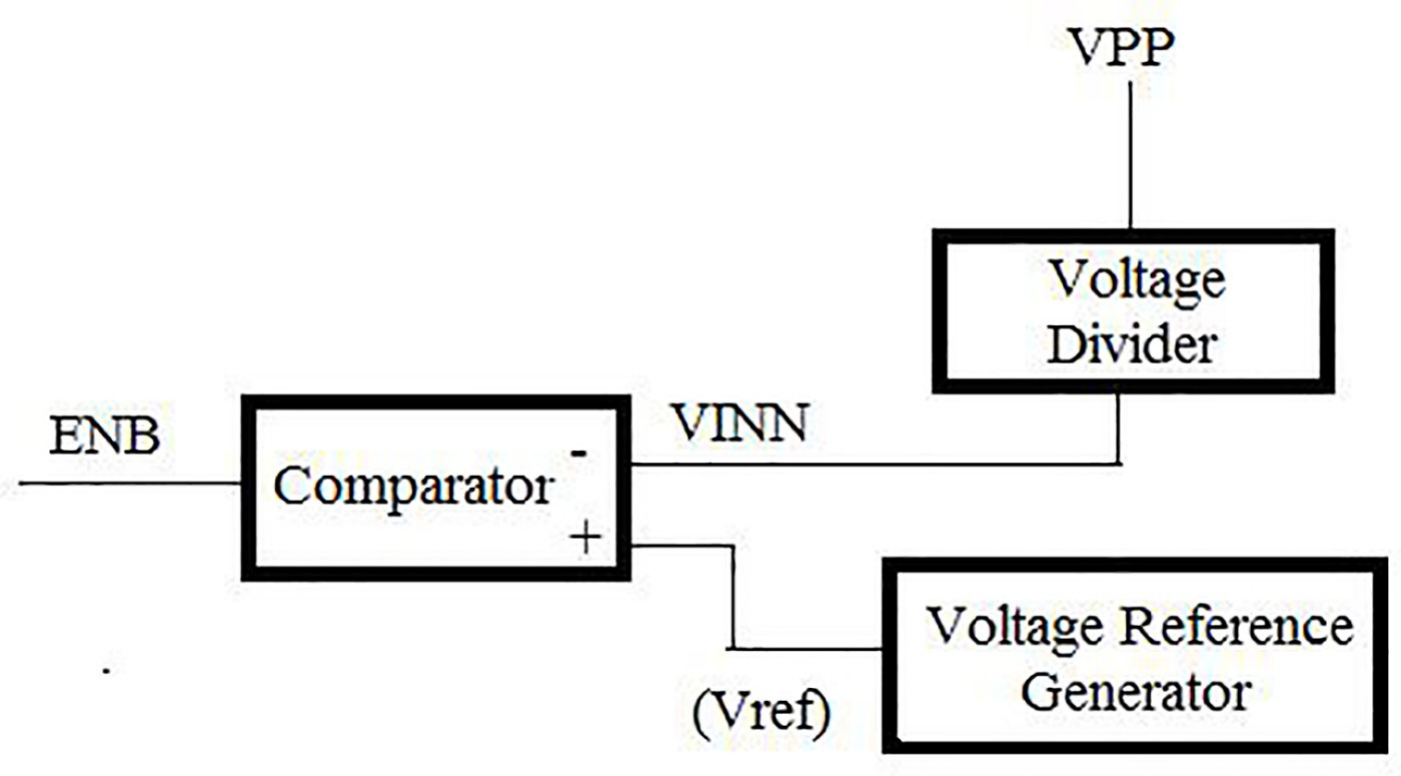
Block diagram of the voltage regulator.

To stabilize the high voltage and reduce power consumption, a voltage regulator is used and illustrated in Fig 4. When the feedback signal produced by the bandgap reference reaches values higher than V_ref_, the comparator and logic control circuit work together to turn off the clock. Similarly, when the feedback signal is lower than V_ref_, the comparator circuit compares the VINP and VINN and generates an output, which is used as a feedback signal for the CSRO circuit to continue driving the clock signal generation scheme. In addition, by using the capacitive voltage divider circuit instead of the resistive divider, the power dissipation reduced significantly throughout the entire HVG generation process.

### Integrated high-voltage generator

**Fig 5:**
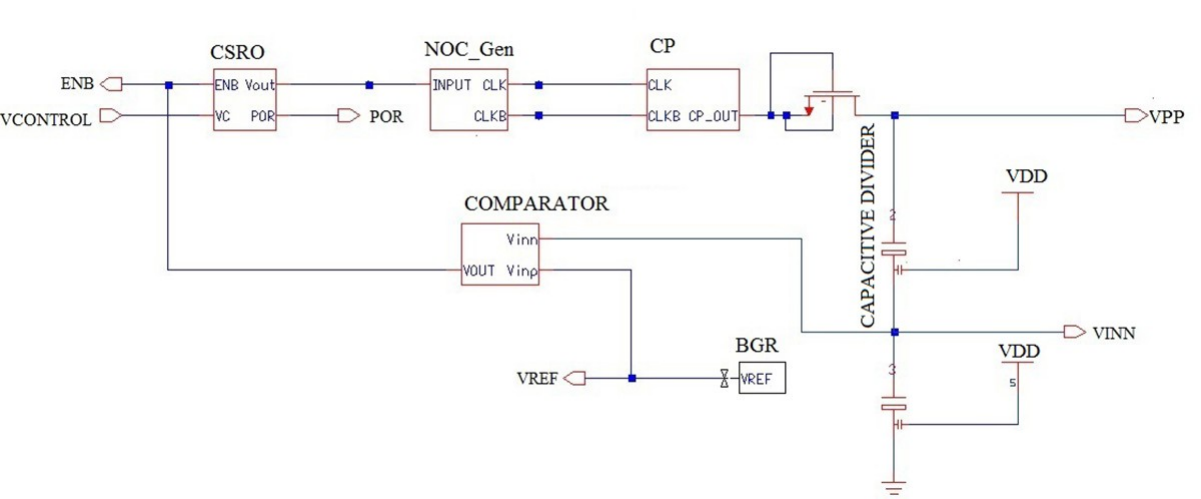
Schematic diagram of the proposed HVG circuit.

Fig 5 shows the schematic diagram of the proposed HVG after the integration of all the modules. In this research, a CSRO output OSC_OUT is used as the input to the NOC_Gen to drive the clock signal and produce the two antiphase clock signals CLK and CLKB. These clock signals are utilized in the proposed CTS CP circuit to complete the charge transfer process at each stage. In this study, the CTS CP circuit is the core circuits used to generate a higher VPP for the entire HVG. However, the major difficulty associated with the design of a CTS CP circuit is the pumping efficiency, which is reduced owing to the large ripple voltages in the case at which the number of pumping stages is increased. Nevertheless, in the EEPROM a large part of the total power is consumed by the HVG circuit, which must be designed properly to meet the demands. Therefore, feedback control/regulator circuits are employed in this design method to reduce the ripple voltage, increase efficiency, and decrease the power dissipation of the HVG. In addition, a diode-connected NMOS transistor is added after the output of the CP circuit, as it will provide one diode drop (V_in_) before the diode-connected NMOS to reduce the ripple from the output voltage VPP. After the diode-connected NMOS stage, a capacitive voltage divider is added to generate the divided voltage VINN for the regulator circuit. Conversely, to provide an internal reference voltage that is independent of the process temperature, a BGR circuit is added in this research to generate a reference voltage VREF that has a constant voltage, regardless of power supply variations or temperature changes. This VREF and VINN acted as the inputs for the comparator circuit to feedback to the output of the CSRO circuit to trigger the oscillation process. The regulation scheme takes advantage of the regulation capabilities of a comparator that is featured in this research study. It is also shown in Fig 5 that the oscillator will start working when the ENB node becomes low, which eventually starts the oscillation process of the CSRO circuit. After reaching a certain voltage, this proposed HVG output voltage VPP will be in the steady-state and will help write/erase operation of the RFID transponder EEPROM.

**Fig 6:**
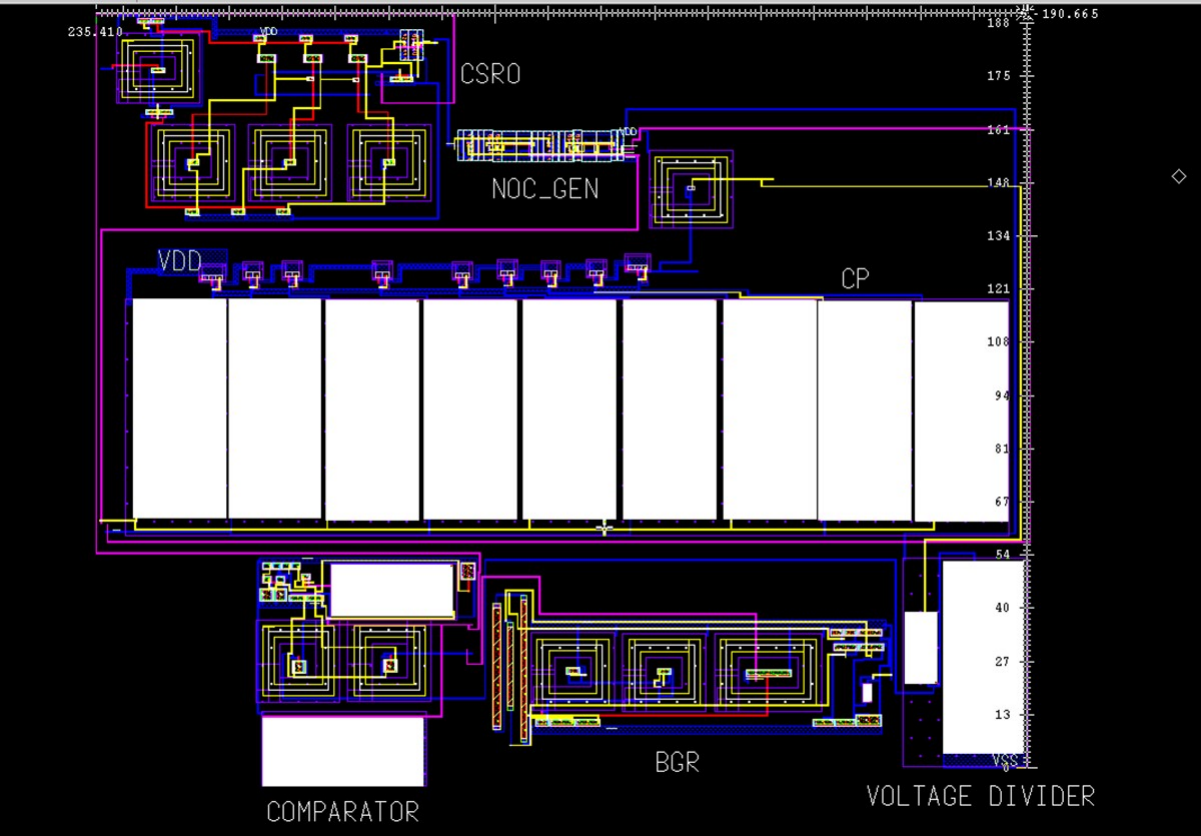
Die layout of the proposed HVG circuit without pads.

**Fig 7:**
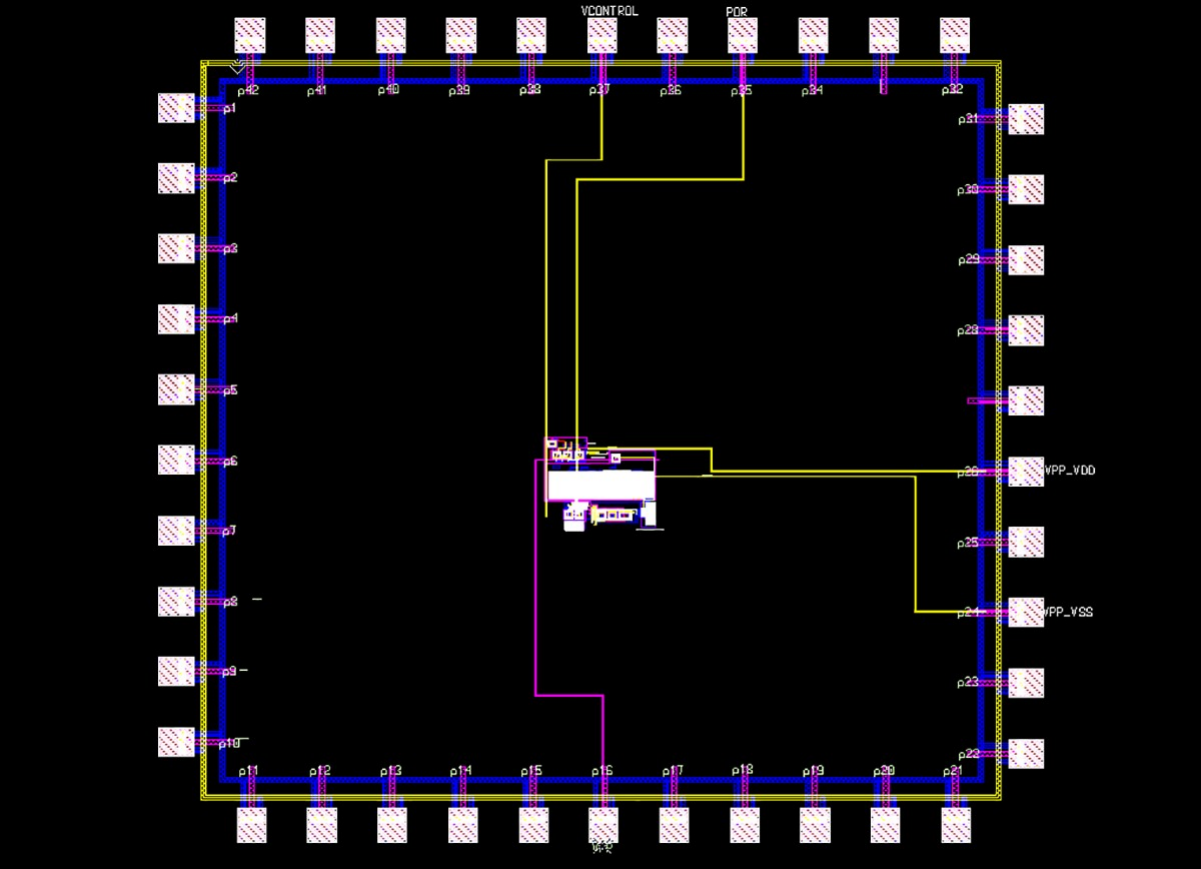
Chip layout of the proposed HVG with input/output pads.

The proposed HVG circuit layout is shown in Fig 6, where the overall design occupies a chip area of 235.41 μm × 190.66 μm, and the chip layout of the proposed HVG with input/output (I/O) pads for fabrication is shown in Fig 7.

## Results and discussion

**Fig 8:**
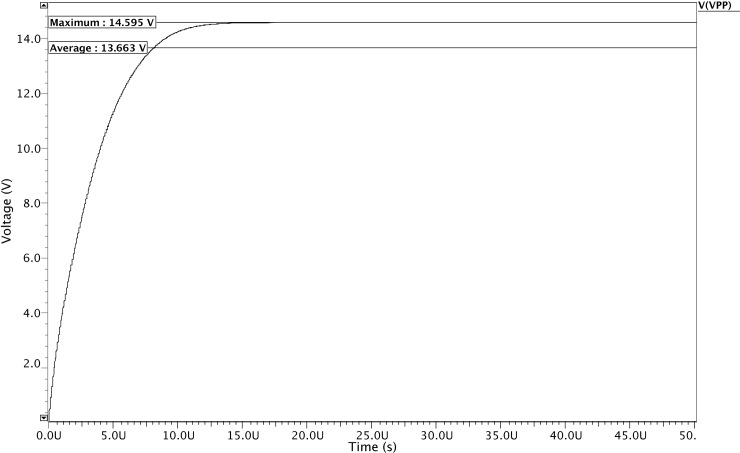
Post layout simulation output for the proposed HVG circuit.

In this section, the overall performance of the proposed HVG circuit is illustrated after integrating all the modules. The proposed HVG is simulated using the ELDOSPICE simulator (Mentor Graphics) in a "Silterra 0.13 μm" CMOS process. After integrating all the modules CSRO, NOC_Gen, CP, and the voltage regulator circuit, a pre-layout simulation result is obtained, as shown in Fig 8, whereby a high VPP of 14.59 V is generated from the proposed HVG for a power supply voltage of 1.8 V.

**Fig 9:**
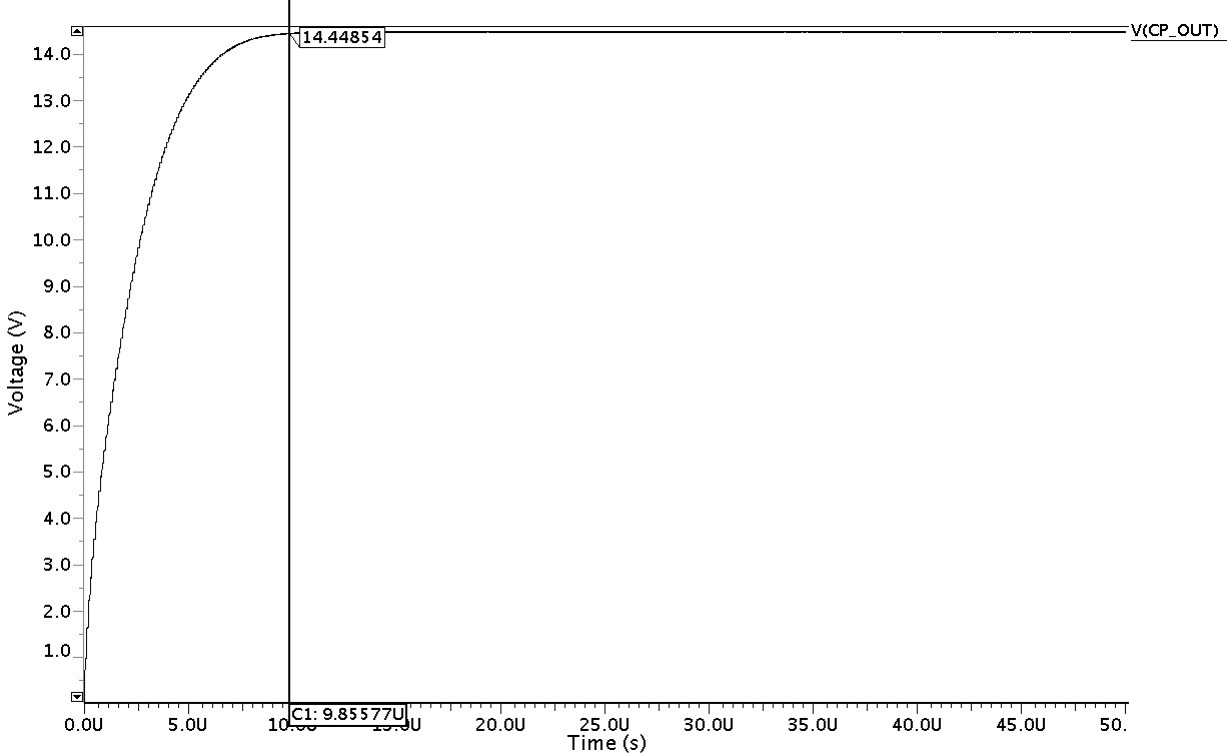
Simulated output ramp-up time for the proposed HVG circuit.

To boost the output voltage, the clock frequency of 11.3 MHz which are generated by the CSRO and the NOC_Gen circuits are used as the input in the charge transfer process of the CP circuit, as shown in Fig 5. Moreover, the reference voltage VREF is found at 1.2 V to continue the voltage regulation process of the proposed HVG. Moreover, the ramp-up time for the overall high-voltage generation process is found to be only 9.8 μs with a load current of only 145.8 nA, as shown in Fig 9. As a result, the integrated HVG consumes only 0.12 mW for a supply voltage of 1.8 V, which is the lowest among those reported in previous research studies.

**Fig 10:**
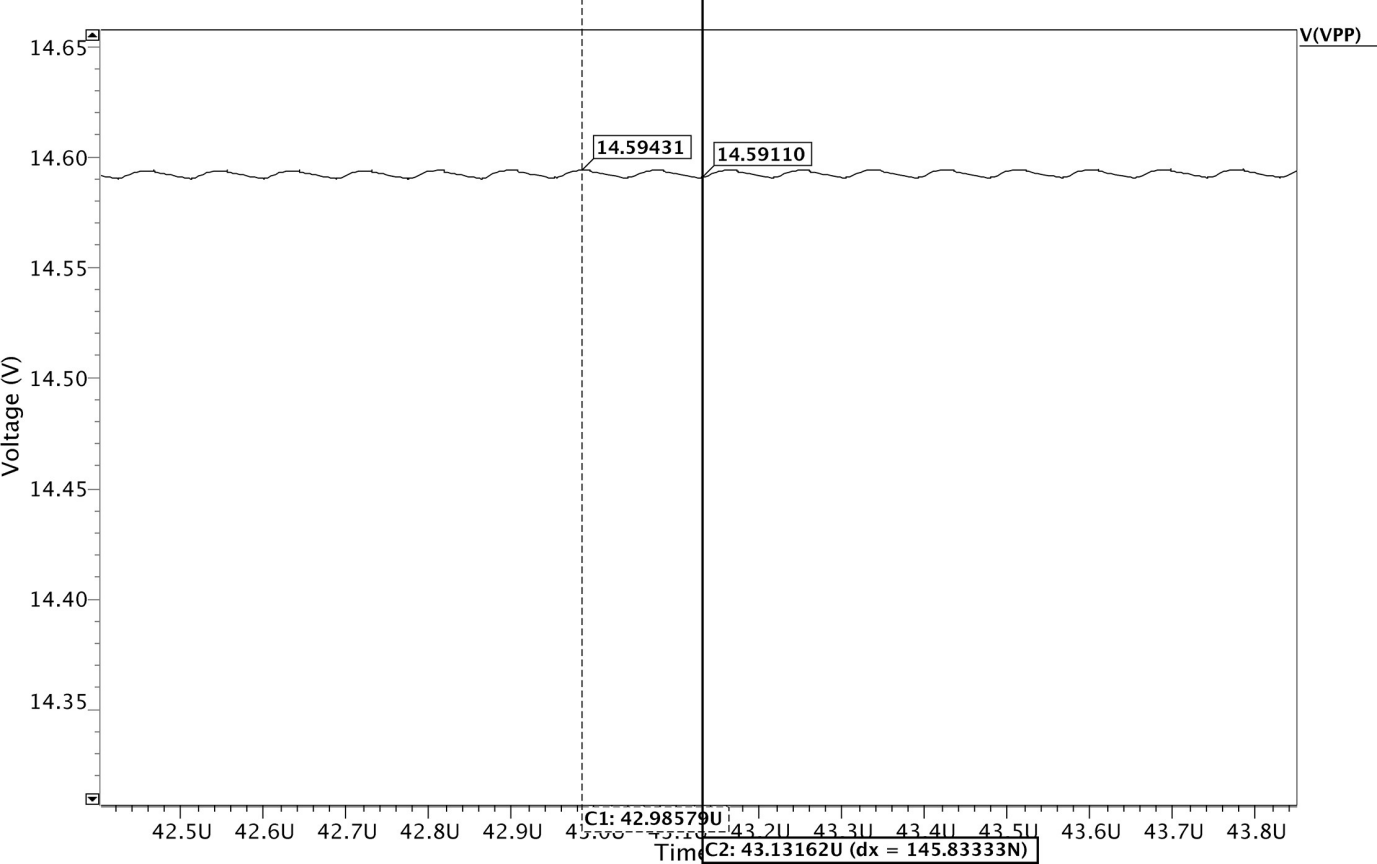
Simulated output ripple voltage of the proposed HVG.

In this study, a voltage regulator (BGR, comparator, and a voltage divider) is required to create a feedback signal to stabilize the generated high voltage VPP to 14.59 V with a small ripple voltage set to <4 mV, as shown in Fig 10. The magnitude of this ripple is lower than those reported in previous research studies. Furthermore, the completed HVG circuit outcomes have been compared with the recently published research studies, which are presented in Table 1.

**Table 1:** Performance comparison of the different HVG circuits.

Parameters	[14]	[18]	[19]	[20]	[21]	[22]	[23]	[24]	[25]	This Work
**Process (μm)**	0.35	0.18	0.35	0.35	0.35	0.13	0.18	0.13	0.18	0.13
**Supply voltage (V)**	1.5	1.8	3.3	2.9–5.5	3	3.3	3.3	1.2	0.55–0.7	1.2 V ~ 1.8
**Frequency (MHz)**	5	-	1.6~5.5	250 KHz	5	400KHz-600 KHz	-	10	0.5–1.8	11.3
**CP method**	Improved CTS	Diode-connected	Diode-connected	Dual-Phase	Dickson	Automatic pumping current control	Phase-shifted clock scheme	Dickson	CP with stage and frequency modulation	CTS
**Output Voltage (VPP)**	13.6	12	4.8 ~ 8.5	5	14	4.5 ~5.5	10.5	7.2	1.1–3.4	9.6 ~ 14.59
**Ripple Voltage (mV)**	21	-	60	-	-	33.8	-	-	-	<4
**Power consumption (mW)**	0.15	-	0.792	0.264	0.8	-	26	-	-	0.12
**Efficiency (%)**	83.3		> 75	-	83	70	69	75	66	88 ~ 90
**Chip area (mm**^**2**^**)**	-	-	-	-	-	0.25	-	0.5	0.17	0.044

**Fig 11:**
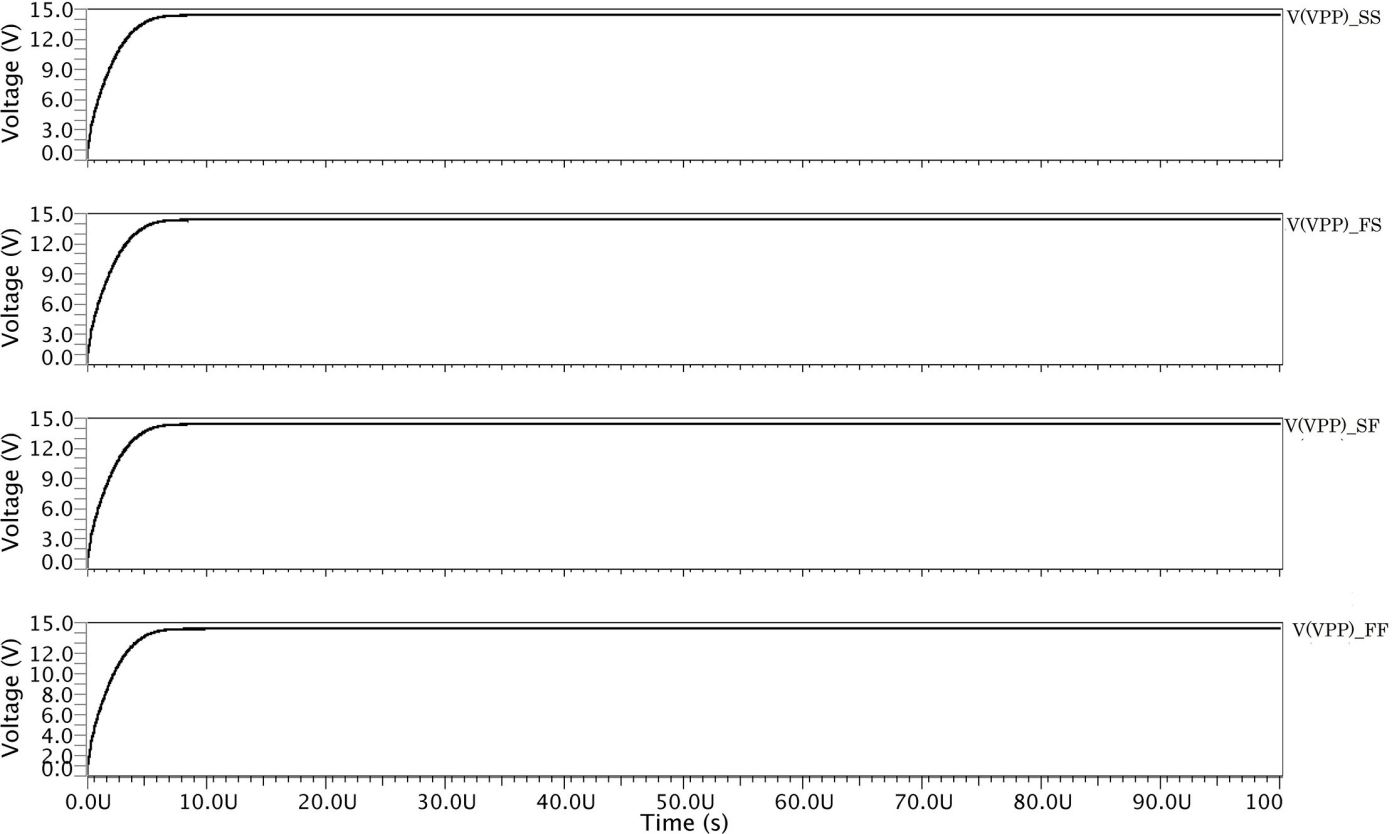
Corner analysis of the proposed HVG circuit.

To verify the external signals, manufacturing tolerances for devices, temperature range, and process variations were tested with a statistical analysis known as corner analysis. Therefore, it is necessary to test the proposed HVG for all the 45 corners and 3 VDD values (1.7 V, 1.8 V, and 1.9 V), 3 temperature, and 5 corners, as shown in Fig 11. From Fig 11, it is revealed that the proposed HVG circuit is able to switch properly at the different corners of the VDD and temperature.

**Fig 12:**
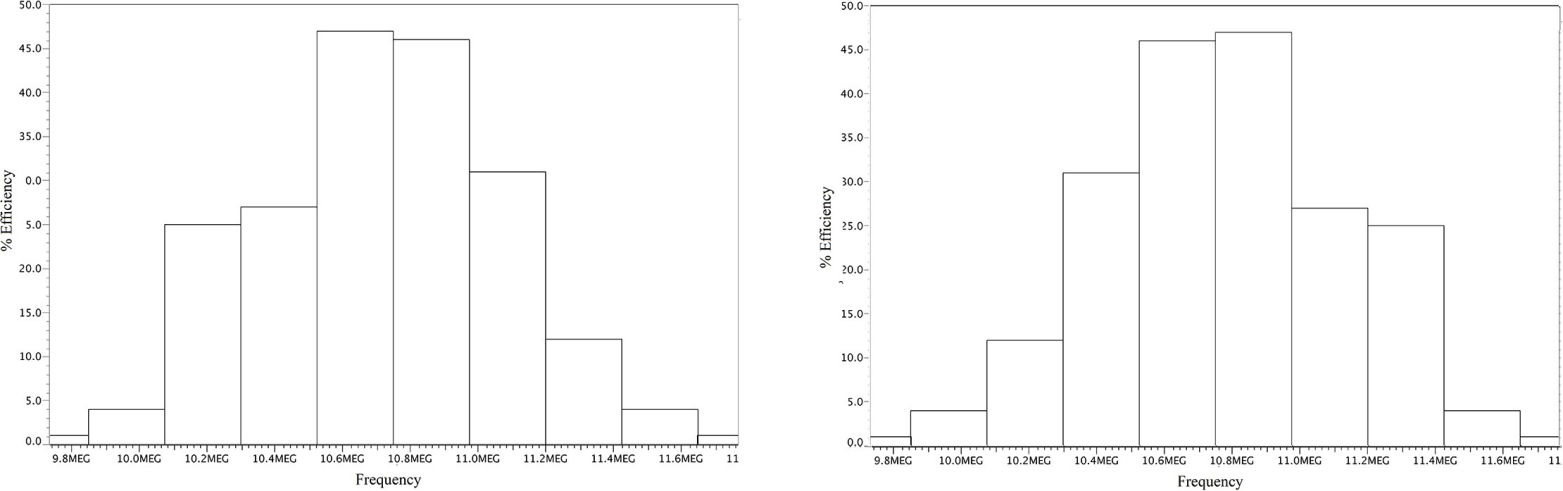
Monte Carlo simulation result for the proposed HVG.

To check the impact of the transistor mismatch during the design, a Monte Carlo simulation was performed with 100 runs for this proposed design, as shown in Fig 12. In addition, this evaluation is able to provide a process variation mismatch.

Table 1 illustrates the performance comparison of the proposed HVG with other recently published research studies [14, 18–25]. Most of the previous research studies have used diode-connected configurations to design the CP circuit, which is the most power-hungry module in HVG [14, 18–19, 21, 23, 25]. Yan et al. [14] utilize the improved CTS method based on diode-connected configuration. In this scheme, a zero Vth MOSFET is employed to overcome the Vth drop at every stage during the charge transfer process. Moreover, this method is able to turn on/off the CTS’s completely, which reduces the feedback current and increases efficiency. However, owing to the voltage loss problem in every stage of the diode-connected configuration resulted in poor output voltage generation with large noise and poor efficiency. Therefore, in this research we propose and present a modified CTS-based CP circuit with a better output voltage with low-ripple and high-pumping efficiency compared to previous research studies [14, 18, 19, 24]. After the successful individual circuit performances, all the modules are integrated to form the proposed HVG circuit. Subsequently, the integrated HVG circuit performances also able to meet the specs with the highest output voltage 14. 59 V compared to previous work [14, 18, 21, 23, 24] and the lowest ripple voltage <4 mV compared to [14, 19, 22]. Conversely, the proposed HVG dissipated only 0.12 mW, which is found to be the lowest among the rated power levels reported in other research studies [14, 19–21, 23]. In this proposed scheme, PMOS transistors were not utilized. Corresponding, this eventually reduces the overall power dissipation of the proposed circuitry and meets the low-power design specification of the intended applications. In addition, the proposed HVG has the highest pumping efficiency of 88 ~ 90% compared to other research studies [14, 19, 21–23]. In this study, the obtained frequency of 11.2 MHz makes the proposed design superior to those reported in [14, 19, 21–25] and adequate for the proposed HVG performance to be compatible with the RFID transponder EEPROM. As the aim of this research was to reduce the power dissipation and the ripple voltage, so it is obvious from Table 1 that the proposed HVG achieved the desired results that were compatible with the RFID transponder EEPROM.

With the development of low-power applications, such as the RFID transponder EEPROM, the internal components, especially the most “power-hungry” HVG module CP, need to dissipate smaller powers while they boost the output voltages. This proposed HVG provides a boosted output voltage that is higher than the voltage of the power supply to meet the requirement of erase/write operations of the RFID transponder’s EEPROM. Obviously, the purpose of this research was to design a low-power and low-ripple HVG circuit with a better pumping efficiency. Therefore, in this research, a three-stage CSRO was used that was able to reduce the power dissipation and the phase noise due to the optimization of transistor sizing and the biasing circuitries. In the HVG, the maximum amount of power was consumed by the CP circuit, as this circuit was utilized to boost up the supply voltage. Therefore, in this research, an eight-stage modified CTS-based CP circuit was proposed which employed triple well NMOS CTSs rather than diode-configured switches [14, 24–25]. In addition, no substrate current was formed throughout the dynamic control process, as all the PMOS transistors were removed from each stage of this proposed design. As a result, the proposed CP circuit produced higher output voltage gains with better reliability. Moreover, the use of pass transistors reduced the voltage loss problems and the reversed charge-sharing problem in the proposed CP circuit. As a result, the overall high-voltage generation process was boosted compared to other research studies [14, 18–25]. Conversely, the use of the NMOS switch between the CP circuit and the voltage regulator reduced the output ripple voltage of the proposed HVG. This ripple voltage is the lowest among the recently reported values in Table 1 [14, 19, 22]. Thus, from the above discussion, it is clear that the proposed HVG meets the challenge of low-power, low-ripple, and high-pumping efficiency requirements, which have become one of the dominant design aspects in CMOS technologies based on low-power applications, like RFID transponders, NVM, and DC-DC converters.

## Conclusions

A fully integrated HVG has been proposed for the RFID transponder EEPROM to perform the write/erase operations using the Silterra 130 nm CMOS process. The power dissipations of the proposed HVG and of almost all of the modules were reduced by optimizing the circuitries, by optimizing the W/L ratio of the transistors, and by avoiding the on-chip passive components. After all the individual performances of the CSRO, NOC-Gen, CP circuits, and the voltage regulator (BGR, comparator and voltage divider) were verified, all the modules were integrated to form the proposed HVG circuit. From the simulated results, it was found that the proposed HVG consumed only 0.12 mW of power and reduced the ripple voltage to <4 mV, which is the lowest among the values reported in other research studies. In addition, the pumping efficiency of the proposed HVG achieved 90% and the boosted output voltage was found to be 14.59 V for a power supply voltage of 1.8 V. This is the highest voltage recorded to-this-date compared to those reported by all the recently published studies in the field. Therefore, the performances of all the modules as well as the integrated HVG are apparently suitable for RFID transponder EEPROMs.
